# Preliminary evaluation of a monovalent antivenom targeting *Cerastes cerastes* envenomatio*n i*n North Africa: feasibility and specificity assessment

**DOI:** 10.3389/fphar.2026.1719611

**Published:** 2026-03-26

**Authors:** Nihal Mesmoudi, Salma Chakir, Khawla Ammouch, Reda Chahir, Hinde Aassila, Abdellah Moustaghfir, Mehdi Karkouri, Naoual Oukkache

**Affiliations:** 1 Laboratory of Venoms and Toxins, Pasteur Institute of Morocco, Casablanca, Morocco; 2 Laboratory of Odontological Biomaterials and Nanotechnology, Faculty of Dental Medicine, Mohammed V University in Rabat, Rabat, Morocco; 3 Laboratory of Agri-Food and Health, Faculty of Science and Technology, Hassan First University of Settat, Settat, Morocco; 4 Laboratory of Pathological Anatomy, University Hospital Center Ibn Rochd, Casablanca, Morocco

**Keywords:** *Cerastes cerastes*, hemorrhagic effects, histological analysis, neutralizing antibodies, snake envenomation

## Abstract

In 2017, the World Health Organization (WHO) recognized snakebite envenomation as a neglected disease yet the therapeutic effectiveness efficacy of available antivenoms remains insufficiently explored. This study offers a detailed analysis of *Cerastes cerastes* venom, focusing its toxicological properties and the development of specific targeted neutralizing antibodies. Through rigorous quality control and comprehensive efficacy testing, the antivenom demonstrated significant neutralizing activity against critical venom components, including hemorrhagic, edema-forming, and myotoxic effects while also mitigating tissue damage. Histological investigations further corroborated the antivenom’s protective capacity. These findings not only underscore the potential of the developed antivenom for clinical use but also provide essential insights for advancing antivenom production, refining its specificity, and enhancing its therapeutic efficacy in managing snake envenomations.

## 1 Introduction

Globally, ophidian envenomation is a major neglected tropical disease, with an estimated 1.8 to 2.7 million snakebite incidents annually and mortality rates ranging from 81,410 to 137,880 deaths worldwide (Mochales-Riaño et al., 2024), including approximately 20,000 in Africa (Farooq et al., 2022). In Morocco, envenomation cases are often underreported due to the lack of reliable notification systems, a common issue in developing countries. Most incidents in Morocco occur in rural areas, where *Cerastes cerastes* (the horned viper) is the most prevalent venomous snake, responsible for 17.5% of cases (Chafiq et al., 2023). This species, adapted to desert ecosystems (Mochales-Riaño et al., 2024), is a primary cause of hemorrhagic syndromes (Aissaoui et al., 2013). It typically measures 35–60 cm, preys on small mammals and birds (Fahmi et al., 2012), and produces a venom rich in phospholipases A2 (PLA2s), snake venom metalloproteases (SVMPs), serine proteases (SVSPs), hyaluronidases, and L-amino acid oxidases (LAAOs) (Bazaa et al., 2005). These proteins contribute to toxic effects through additive or synergistic mechanisms, each targeting specific cells and tissues (Abdelkafi-Koubaa et al., 2021). The World Health Organization (WHO) recognizes antivenoms as a priority, listing them as Essential Medicines in 2007 (S. WHO Expert Committee on the Selection and Use of Essential Medicines, 2007). Standard treatment relies on immunoglobulins derived from hyperimmunized animals such as horses or sheep (Bailon Calderon et al., 2020). Antivenoms vary in composition, including whole IgG molecules, F (ab')_2_ fragments (pepsin-digested), and Fab fragments (papain-digested), with effectiveness dependent on the immunochemical diversity of snake venoms (Gutiérrez et al., 2006). The only available antivenom in Morocco, Inoserp MENA (Chafiq et al., 2023), targets multiple species but has limited efficacy for some, including *C. cerastes* (Khourcha et al., 2023). A key challenge in antivenom production is venom origin; many formulations use mixtures of uncertain provenance rather than venoms from local species. Given the similarity of *C. cerastes* venom across North Africa, locally sourced antivenoms would significantly improve treatment efficacy (Fahmi et al., 2012; Khourcha et al., 2023). Although polyvalent antivenoms remain the most widely recommended and practical option for the management of snakebite envenomation, the objective of the present study is to develop a species-specific antivenom directed against *Cerastes cerastes* venom, which can be considered monovalent due to its specificity toward this species. This approach is justified by the predominant role of *C. cerastes* in envenomation cases in Morocco and by the documented inter-population variability of its venom, which may limit the effectiveness of currently available polyvalent antivenoms. Our previous work (Khourcha et al., 2023), demonstrated that the antivenom currently commercialized in the country exhibits a suboptimal neutralization capacity against local venoms. In this context, the present study highlights the importance of developing targeted antivenoms adapted to venoms from local snake populations. It also proposes an innovative strategy consisting of producing several monovalent antivenoms specific to the most medically relevant species in each region, followed by their rational combination to generate optimized regional antivenom formulations. Such an approach could significantly improve clinical efficacy while taking into account venom variability and region-specific needs. Accordingly, our study focuses on the toxicological characterization of *C. cerastes* venom and on the development of a monovalent antivenom capable of neutralizing its hemorrhagic, edematous, and myotoxic effects, with histological analyses providing confirmation of its protective efficacy. By developing a species-specific antivenom, this work aims to improve clinical outcomes and reduce snakebite-related mortality in North Africa.

## 2 Materials and methods

### 2.1 Animals and ethical statement

All animal experiments were conducted in accordance with the recommendations of the World Health Organization (WHO) and the European Directive 2010/63/EU on the protection of animals used for scientific purposes.

Rabbits and male Swiss mice (18–22 g) were obtained from the Institut Pasteur of Morocco’s animal facility. They were housed under standard conditions (22 °C ± 2 °C) with a 12-h light/dark cycle and had unrestricted access to food and water.

### 2.2 Venom of *Cerastes cerastes*

Twenty specimens of *Cerastes cerastes*, from juveniles to adults, were collected in Fask, Morocco, a high-risk envenomation area 25 km from Guelmim. This ensured the venom mixture contained all antigenic variations across populations. Venom was manually extracted by fang stimulation. After extraction, the venom was diluted in cold sterile water to protect protein integrity, facilitate handling, and ensure efficient separation of soluble and insoluble components, then centrifuged at 13,000 g (4 °C, 15 min), lyophilized, and stored at −20 °C until use. Consistent venom batches were used for all analyses. Protein concentration was determined via UV absorption at 280 nm using a quartz cuvette (1 cm path length), with 1 absorbance unit corresponding to 1 mg/mL of protein. Measurements were taken using a UV/Visible spectrophotometer (Jenway, Series 67 Model 6715) (Stoscheck and Deutscher, 1990).

### 2.3 SDS-PAGE and densitometric analysis

Venom proteins were analyzed under reducing conditions (5% β-mercaptoethanol). A 20 μg sample was loaded onto a 12% SDS-polyacrylamide gel, with molecular weights estimated using markers (14.4–97 kDa). Samples were heated (95 °C, 5 min) before electrophoresis (120 V, 60 min). Gels were stained with Coomassie blue R-250 and analyzed for protein band intensity and antibody purity.

### 2.4 Median lethal dose (LD_50_) determination

The median lethal dose (LD_50_) of the venom was determined in accordance with the World Health Organization (WHO) guidelines (Gutiérrez et al., 1986). Groups of six male Swiss mice (18–22 g) were administered increasing doses of venom by intraperitoneal (IP) or intravenous (IV) injection. Mortality was monitored over a 24-h period following administration. The LD_50_ values were calculated using GraphPad Prism 7 software by nonlinear regression analysis employing a four-parameter logistic dose–response model, with the lower and upper asymptotes constrained to 0% and 100% mortality, respectively. This approach allowed accurate estimation of the venom’s median lethal dose.

### 2.5 Hemorrhagic activity

Hemorrhagic activity of *C. cerastes* venom was assessed using the WHO mouse skin assay (Gutiérrez et al., 1986). Swiss mice (18–22 g; n = 3/group) received intradermal injections of venom (≤10 µg in 100 µL saline) or PBS (control). After 2 h, mice were euthanized, and skin lesions were excised and measured using a caliper. The minimum hemorrhagic dose (MHD) was defined as the lowest venom dose producing a hemorrhagic lesion ≥10 mm in diameter. Tissue samples were fixed in 10% neutral-buffered formalin for histological analysis.

### 2.6 Edema-forming activity

Edema activity was evaluated using a mouse footpad assay (Gutiérrez et al., 1986). Swiss mice received a subcutaneous injection of venom (0.5–20 µg) in one footpad and 0.9% NaCl (control) in the other. Edema was monitored over 180 min. The minimum edema-forming dose (MED) was the smallest venom dose causing a ≥30% foot size increase.

### 2.7 Myotoxic activity

Myotoxicity was assessed via histology and creatine kinase (CK) activity after injecting a sublethal venom dose (20 µg/mouse) into the gastrocnemius muscle of Swiss mice. Controls received saline. Three hours post-injection, blood samples were collected, plasma was separated (1800 g, 10 min, 4 °C), and Ck activity was measured. Muscles were fixed, sectioned (5 µm), and stained for histological evaluation.

### 2.8 Tissue alteration

The toxic effects of *C. cerastes* venom on the heart and lungs were evaluated histologically. Swiss mice were divided into two groups (n = 2/group): a control group receiving intraperitoneal (IP) saline (0.9% NaCl) and a venom-treated group receiving an IP injection of a half-lethal dose (½ LD_50_) of venom (500 µL). Following euthanasia, organs were excised, fixed in 10% neutral-buffered formalin for 24 h, dehydrated, embedded in paraffin, sectioned at 4 μm, and stained with Mayer’s hematoxylin for microscopic examination.

### 2.9 Neutralizing antibody production

Rabbits were immunized with *C. cerastes* venom to produce antibodies. Plasma was collected, and antibodies were purified via affinity chromatography (Morais and Massaldi, 2006).

### 2.10 Antigen preparation and immunization

Venom samples from *C. cerastes* were filtered through 0.22 µm membranes and used for antivenom production. Male rabbits (2–2.5 kg), including one control animal, were immunized for antibody production. For antigen preparation, venom was emulsified with an equal volume of Freund’s complete adjuvant for the primary injection, followed by emulsification with Freund’s incomplete adjuvant for the second and third injections. Increasing venom doses were administered subcutaneously into the cervical region near the lymph nodes according to the immunization schedule (Table 1). Immune response induction was monitored by lymph node formation following each injection (Russell et al., 1985; Zarzosa et al., 2024).

**TABLE 1:** Immunization schedule for rabbits against *C. cerastes* venom.

Number of bleedings	Days	Venom dose (µg/rabbit)	Adjuvant	Volume injected (ml/rabbit)	Number and route of injection
1	0	20	CFA (1.3)	1	SC (6)
2	07	20	IFA (1.3)	1	SC (3)
3	14	50	IFA (1.0)	1	SC (3)
4	21	100	IFA (1.3)	1	SC (3)
5	28	150	NaCl 0.9%	1	SC(3)
6	35	200	NaCl 0.9%	1	SC(3)
7	42	Blood collection			

### 2.11 Control of antibody production: immunodiffusion assay

Radial immunodiffusion was used to monitor antivenom production and assess its immunoreactivity to raw venom. Slides with 1% agarose in sodium barbital buffer (pH 8.6) or 0.85% NaCl were prepared. Wells were loaded with 15 µL of a 5 mg/mL *C cerastes* venom solution and antivenom solution. Slides were incubated at 37 °C for 24–48 h, washed with 0.15 M NaCl for 72 h, dried at 37 °C, and stained with Coomassie Brilliant Blue R.

### 2.12 Blood collection

Blood samples were collected from the marginal ear vein under local anesthesia. In stress-induced vasodilation cases, central vein access was considered. Serum antibody concentration was determined via radial immunodiffusion. Whole blood samples were drawn into 3.5 mL dry tubes, centrifuged at 3,000 rpm for 10 min, and sera stored at −20 °C.

### 2.13 Purification of neutralizing antibody: affinity chromatography

Immunoaffinity chromatography was used for purification. Sepharose 4B gel was activated with cyanogen bromide, swollen in 1 mM HCl, and blocked. Antivenom (3.3 mL serum) was mixed with 0.1 M sodium bicarbonate (pH 8) and incubated overnight at 4 °C. After centrifugation, the supernatant was mixed with gel and incubated for antigen binding, monitored by absorbance. The gel was activated with Tris (1 M, pH 8) before venom loading. Eluted fractions were collected in Tris-HCl buffer (1 M, pH 8), and antibody concentration was assessed at 280 nm.

### 2.14 Quality control of neutralizing antibody

#### 2.14.1 Protein concentration

Determined via Bradford assay using a six-point calibration curve (0–10 μg/μL) with BSA standard. Samples were diluted, incubated with Bradford reagent, and absorbance measured at 595 nm (Bradford, 1976).

#### 2.14.2 Purity assessment

The purity and relative abundance of the neutralizing antibodies were assessed by densitometry using the Gel Analyzer software, following antibody purification from serum via affinity chromatography.

#### 2.14.3 Specificity assessment

The presence of specific antibodies against the venom was assessed by the Ouchterlony double-immunodiffusion method (Ouchterlony, 1962). A 1% agarose gel in 0.15 M NaCl (pH 7.4) was cast on glass plates, and wells were cut for antigen and antibody solutions. Equal volumes (20 µL) of venom and antibody solution were added to their respective wells and incubated in a humid chamber at room temperature for 48–72 h. The appearance of visible precipitin lines indicated antigen–antibody interactions.

#### 2.14.4 Efficacy assessment

##### 2.14.4.1 Median Effective Dose (ED50)

Antivenom neutralization was tested by pre-incubating 3LD50 venom with varying antivenom doses (100–350 µL) and injecting intraperitoneally into mice (n = 4 per dose). Survival rates after 24 h determined ED50 and neutralization potency (Morais et al., 2010).

##### 2.14.4.2 Neutralization of hemorrhagic activity (MHD-ED50)

Using WHO guidelines (WHO, 2025), 2MHD venom was incubated with antivenom and injected intradermally into mice. Hemorrhagic lesion diameters were measured to determine MHD-ED50.

##### 2.14.4.3 Neutralization of edema-forming activity (MED-ED50)

Venom-antibody mixtures were injected into mouse footpads, and edema reduction was analyzed. MED-ED50 was calculated as the venom/antivenom ratio reducing edema by 50% (Gutiérrez et al., 1986).

##### 2.14.4.4 Neutralization of myotoxic activity

Mice received venom-antibody mixtures intramuscularly. Plasma CK levels and muscle histology confirmed myotoxicity neutralization.

##### 2.14.4.5 Neutralization of venom-induced histopathological alterations

Mice received a sublethal venom dose, followed by antivenom (ED50). Control mice received saline. After 4 h, organs were fixed in 10% formaldehyde.

#### 2.14.5 Histological studies

The collected organs were immersed in a 10% formalin solution for 24 h. Subsequently, the tissues were dehydrated through a series of ethanol concentrations, clarified with xylene, embedded in paraffin, and sectioned at 4 μm thickness. These sections were then stained with hematoxylin and eosin for visualization under a light microscope.

### 2.15 Statistical analysis

The tests were conducted at least three times, with data presented as means ± standard deviations (SD). Significant differences among all experimental groups were assessed using a one-way ANOVA in GraphPad Prism 7.0 software. Statistical significance was determined at the following levels: p > 0.05 (ns), p < 0.05 (*), p < 0.01 (**) and p < 0.001 (***). The LD50 of the venoms and ED50 of the antivenoms were calculated using non-linear regression (variable slope) in GraphPad Prism 7.0 software. For comparative analysis, ED50 values of the antivenoms were normalized (n-ED50) based on their respective protein content and expressed as the amount of venom neutralized per Gram of antivenom protein (mg/g).

## 3 Results

### 3.1 SDS-PAGE and densitometric analysis

**FIGURE 1:**
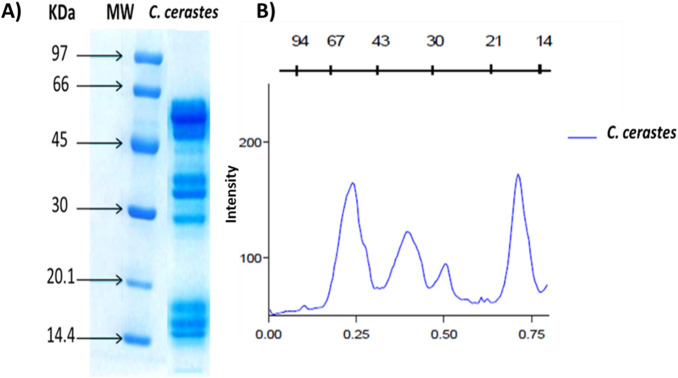
**(A)** 12.5% SDS-PAGE Analysis (12.5%) of *Cerastes cerastes* Venom (CcV): Line 1 corresponds to *C. cerastes* Venom (20 μg Protein), and Line 2 corresponds to Molecular Weight Markers (MW); **(B)** Densitometric Analysis of Protein Bands, Corresponding Quantification of the Venom Protein Profile.

SDS-PAGE analysis of crude *C. cerastes* venom revealed the presence of multiple distinct protein bands. Densitometric analysis indicated that proteins within the 66–45 kDa and 21–14 kDa molecular weight ranges constituted the majority (∼60%) of the total protein content. In contrast, proteins in the 43–30 kDa and 30–25 kDa ranges were present at lower abundance, as reflected by their comparatively weaker band intensities (Figure 1).

### 3.2 Determination of the median lethal dose (LD_50_)

The median lethal dose (LD_50_) of *C. cerastes* venom was determined via two administration routes: intravenous (IV) and intraperitoneal (IP). The LD_50_ values were 18 μg/kg (15.4–22 μg/kg) for IV injection and 36.30 μg/kg (30.98–40.57 μg/kg) for IP injection (Table 2). Notably, the LD_50_ for IV administration was approximately half that of the IP route, indicating a significantly higher lethality when the venom is introduced directly into the bloodstream. This difference may be attributed to the higher molecular weight (MW) of certain venom components, which, when injected intraperitoneally, require more time to enter systemic circulation due to slower absorption from the peritoneal cavity (Table 1).

**TABLE 2:** Determination of the Median Lethal Dose (LD_50_) of Cerastes cerastes venom via Intraperitoneal (IP) and Intravenous (IV) routes, calculated by non-linear regression using GraphPad Prism 7.

Dose unit	*IV*	*IP*	*RATIO*
µg/mouse	18 (15,4–22)	36.30 (30,98–40.57)	2,02
µg/g	0,9 (0,83–1,09)	1.815 (1,549–2,028)	

### 3.3 Haemorrhagic activity assessment

**FIGURE 2:**
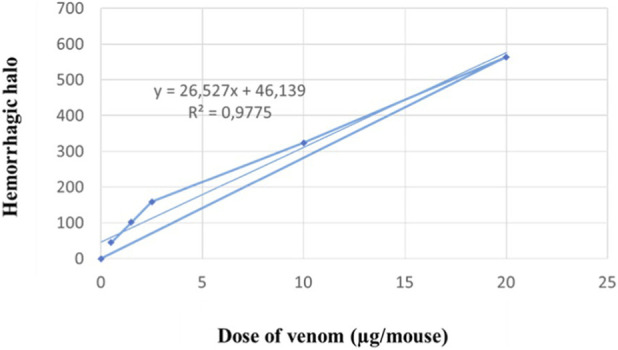
Dose-response curve of hemorrhagic activity induced by various concentrations of *Cerastes cerastes* venom in mice. The graph shows the mean of hemorrhagic halo, calculated using graph paper ±SD (standard deviation) (n = 4). Linear regression of hemorrhagic halos in relation to venom dose is shown (r = 0.9881 for *C. cerastes*).

The haemorrhagic activity of *C. cerastes* venom was evaluated by determining the Minimum Haemorrhagic Dose (MHD), based on the lesion diameter at 50% incidence. *C. cerastes* venom induced a significant, dose-dependent haemorrhagic effect, as evidenced by the progressive increase in lesion size (Figure 2). The venom exhibited a notable haemorrhagic response, with a lesion size of 0.57 ± 0.23 µg.

Histological analysis of venom-injected skin sections revealed extensive tissue damage, extending from the epidermis to the hypodermis. In contrast, control mice injected with physiological saline showed normal histological features, including an intact epidermis, dermis, and hypodermis.

**FIGURE 3:**
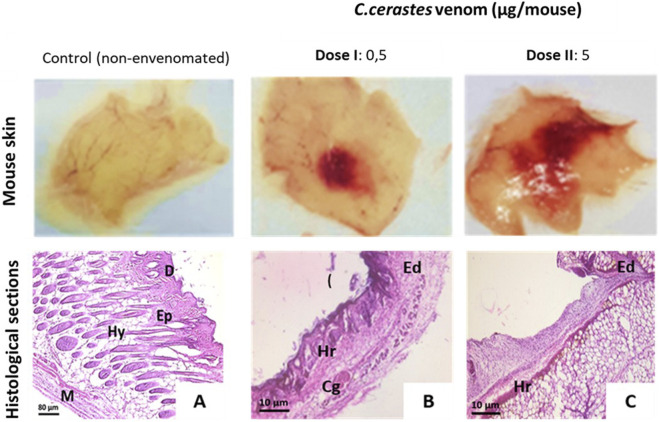
Histopathological analysis of mouse skin. Macroscopic and microscopic observations of mouse skin in panels **(A–C)**. The control group **(A)** shows normal skin structure, with intact dermis, epidermis, and hypodermis. Skin injected with 0.5 µg of *Cerastes cerastes* venom **(B)** exhibits edema, hemorrhage, and cellular degradation. Skin injected with 5 µg of venom **(C)** demonstrates extensive hemorrhage, epidermal desquamation, and hypodermal damage. D; Dermis, Ep; Epidermis, Hy; Hypodermis, Mu; Muscle, Ed; Edema, Hr; Hemorrhage, Cg; Cellular degradation.

Venom-induced lesions were dose-dependent and characterized by edema, ranging from mild (0.5 µg) to moderate (5 µg), with superficial hemorrhages at lower doses (0.5 µg) and deep hemorrhages at higher doses (5 µg). Additionally, vascular congestion and vessel dilation were observed, as illustrated in Figure 3.

### 3.4 Edema-forming activity

**FIGURE 4:**
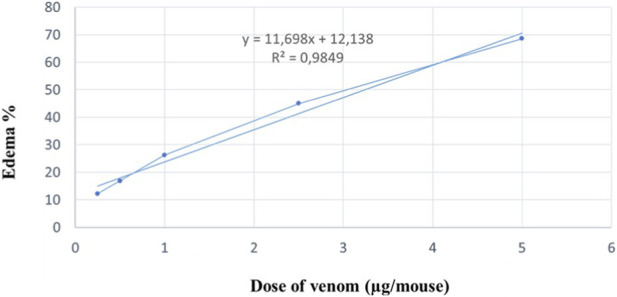
Dose-response curve of edema-forming activity induced by different concentrations of *Cerastes cerastes* venom in mice. The graph shows the percentage increase in the weight of venom-injected footpads compared to the saline-injected footpads. Values represent the means ± SD (standard deviation) (n = 4). Linear regression of venom doses in relation to the percentage of edema (%) is shown (r = 0.9849 for *C. cerastes*).

The edematogenic potential of *C. cerastes* venom was assessed by determining the Minimum Edema-Forming Dose (MED) in a homogeneous population of mice (20 ± 2 g). The results demonstrated a dose-dependent edema-forming activity, with a significant edematogenic response. The MED value was determined to be 0.50 ± 0.22 μg (Figure 4).

### 3.5 Histological analysis of edema formation

**FIGURE 5:**
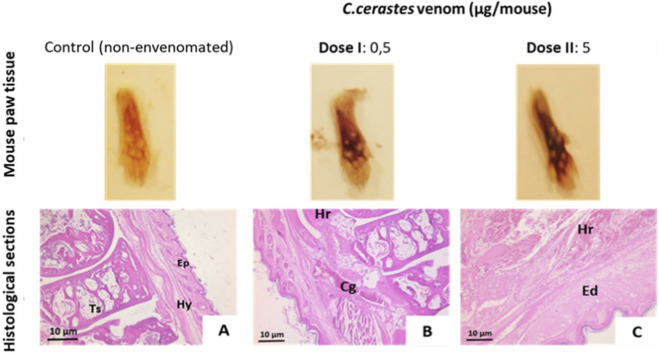
Histopathological analysis of mouse paw tissue following envenomation by *Cerastes cerastes* venom. Macroscopic and microscopic observations reveal the progression of tissue alterations. The control group shows normal epidermal (Ep), dermal (D), and hypodermal (Hy) structure. In contrast, tissue exposed to 0.5 µg of venom exhibits moderate haemorrhage (Hr), cellular degradation (Cg), and hypodermal changes, while tissue exposed to 5 µg demonstrates severe edema (Ed), pronounced hemorrhage (Hr), and extensive structural damage. **(A)** Control group (non-envenomated); **(B)** Dose I (0.5 μg); **(C)** Dose II (5 μg).

Histological examination of the left hind paws injected with physiological saline revealed normal tissue morphology, displaying the three primary skin layers: epidermis (Epi), dermis (Der), and muscle (Mus). In contrast, sections of the right hind paws injected with *C. cerastes* venom exhibited edema, hemorrhage, and inflammation, primarily affecting the dermal and muscular layers. The severity of these effects increased in a dose-dependent manner. A venom dose of 5 µg induced pronounced edema, characterized by discontinuous detachment of the epidermis from the dermis, hemorrhagic areas, and moderate vascular congestion (Figure 5).

### 3.6 Myotoxic activity

The myotoxic activity of *C. cerastes* viper venom was evaluated through both *in vivo* and *in vitro* studies, examining tissue alterations and measuring serum CK levels in envenomed mice.

In the *in vitro* study, blood samples collected three hours after intramuscular injection of a sublethal venom dose showed a significant increase in plasma CK levels compared to control mice (379.667 ± 55.33 IU/L), with CK levels in *C. cerastes*-envenomed mice reaching 1986.4 ± 201.18 IU/L, indicating substantial muscle damage.

**FIGURE 6:**
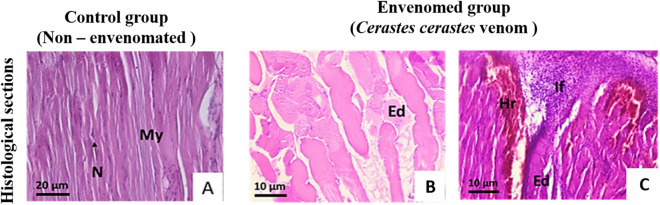
Histopathological analysis of gastrocnemius muscle in mice following envenomation by *Cerastes cerastes* venom. Microscopic evaluation of the gastrocnemius muscle demonstrates significant pathological changes. The control group **(A)** exhibits normal myofibril (My) structure and nuclei (N). In envenomed mice, muscle sections reveal hemorrhage (Hr) and early signs of congestion **(B)**. Tissue exposed to venom demonstrates pronounced edema (Ed) **(C)**. My: Myofibril, N: Nuclei, Hr: Hemorrhage, Ed: Edema.

In the *in vivo* study, notable pathological alterations were observed in the muscles of envenomed mice (Figure 6). Compared to control mice (Figure A), histological sections of the muscles from treated mice revealed extensive hemorrhagic effects with red blood cell extravasation, marked interstitial edema, and vascular congestion. Additionally, a polymorphous inflammatory infiltrate composed of lymphocytes, neutrophils, and macrophages was also observed. These findings highlight the potent myotoxic effects of *C. cerastes* venom.

### 3.7 Tissue alterations

**FIGURE 7:**
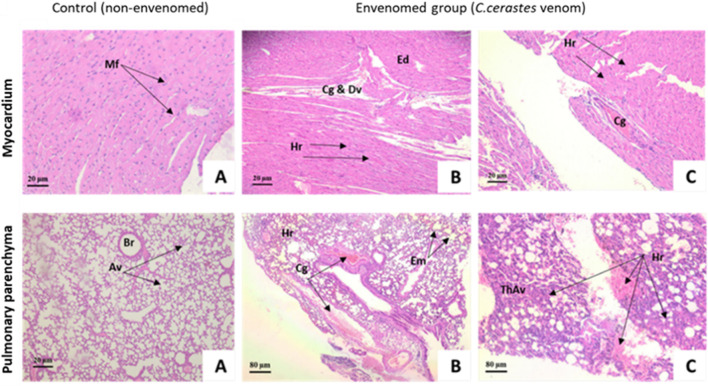
Histopathological examination of the myocardium and pulmonary parenchyma in mice envenomed with Cerastes cerastes venom. Histological analysis of the myocardium in the control group **(A)** revealed normal myocardial fibers (Mf) without visible pathology, whereas envenomed mice **(B,C)** displayed edema (Ed), congestion (Cg), damaged vessel walls (Dv), and extensive hemorrhage (Hr). Examination of the pulmonary parenchyma showed normal bronchioles (Br) and alveoli (Av) in the control group **(A)**, whereas envenomed mice **(B,C)** exhibited severe congestion (Cg), hemorrhage (Hr), early emphysema (Em), and thickened alveolar walls (ThAv), reflecting significant damage caused by venom exposure.

The cardiac muscles of the control mice displayed the typical characteristics of cardiac muscle fibers, each surrounded by an endomysium of delicate connective tissue and a rich capillary network (Figure 7A). The lung tissues of the control mice (Figure 7A) exhibited normal, compact organization, consisting of bronchi, bronchioles, and sac-like specialized structures called alveoli, composed of surface epithelium and blood vessels. In contrast, mice envenomed by the venom of the C. cerastes viper showed alterations in the size and shape of the examined organs, specifically the heart (Figures 7B,C) and lungs (Figures 7B,C). Cardiac level: In comparison with the control group (Figure 7A), histological analysis of the cardiac tissue in mice envenomed by C. cerastes venom revealed a slight loss of myocardial fiber striations, cellular hypertrophy, severe interstitial edema between fibers, as well as congestion and dilation of blood vessels filled with agglutinated red blood cells (Figures 7B,C). Pulmonary level: Compared to the control group (Figure 7A), analysis of the lung tissue in mice envenomed by C. cerastes venom showed significant hemorrhaging in the interstitial space, causing dilation of the inter-alveolar septa. This contributed to inflammatory infiltrates, thickening of the alveolar walls, and bronchial dilation. The presence of emphysematous areas and vascular congestion was also observed (Figures 7B,C).

### 3.8 Control of antibodies production: immunodiffusion assay

**FIGURE 8:**
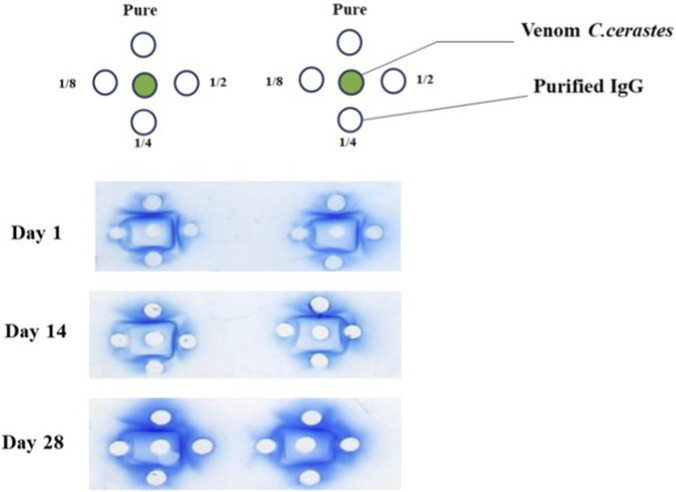
Double immunodiffusion assay illustrates the precipitation arcs formed by the interaction between *Cerastes cerastes (Cc)* venom (in the central well) and the neutralizing antibodies of rabbit plasma (in the peripheral wells), thereby confirming the production of antibodies. **(A)** Peripheral wells containing increasing dilutions of rabbit plasma collected on day 42 after the first immunization.

The immunodiffusion test revealed the presence of multiple antibodies in the sera of immunized rabbits, indicated by the formation of precipitation arcs resulting from antigen-antibody interactions. The venom of *Cerastes cerastes* elicited a robust immune response, with peak antibody production occurring 26 days after the booster. This response continued to escalate beyond 31 days, emphasizing the importance of monitoring antibody production over time to determine the optimal immune response. Based on these findings, blood collection should be performed after week 6, when antibody production reaches its peak (Figure 8).

### 3.9 Purification of neutralizing antibody: affinity chromatography

**FIGURE 9:**
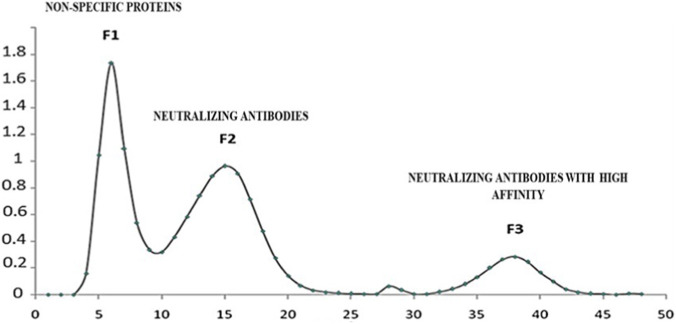
Immunodiffusion test illustrating the precipitation arcs resulting from the interaction between the Cerastes cerastes venom (in the central well) and the neutralizing antibodies of the rabbit plasma (in the peripheral wells), indicating the production of antibodies.

Neutralizing antibodies against *Cerastes cerastes* venom were purified from the serum of immunized rabbits using affinity chromatography, yielding three distinct fractions. Fraction 1 (F1) contains non-specific plasma proteins that do not bind to *Cerastes cerastes* venom. Fraction 2 (F2) includes specific antibodies with moderate affinity for the venom, contributing to the neutralization of its toxic components. Fraction 3 (F3) consists of highly specific neutralizing antibodies with strong affinity for venom toxins, demonstrating enhanced binding capability and likely playing a crucial role in venom neutralization (Figure 9).

### 3.10 Quality control of neutralizing antibody

The concentrations of plasma and purified serum were 9.03 g/mL and 7.01 g/mL, respectively, with an overall yield of 7.53%. These values indicate a successful purification process, ensuring a high concentration of neutralizing antibodies in the final product (Table 3).

**TABLE 3:** Quantification of neutralizing antibody concentration in plasma and purified serum.

Sample type	Concentration g/mL	Volume ml	Quantity g	Yield %
Plasma	9,03	500	46,5	100
Purified serum	7,01	50	3,5	**7,53**

Bold values indicate the yield (%) of purified IgG relative to the initial amount present in plasma.

### 3.11 Evaluation of the purity of the neutralizing antibody

**FIGURE 10:**
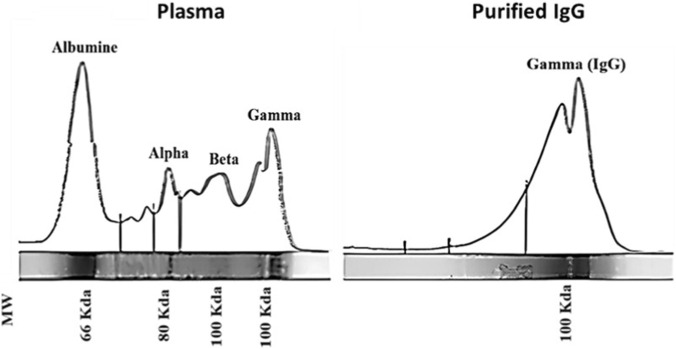
Purification and evaluation of immunoglobulin g (IgG) from plasma via densitometric analysis.

The protein profiles of crude plasma and antibody fractions obtained after affinity chromatography were evaluated by electrophoretic densitometry (Figure 10). The crude plasma exhibited multiple peaks corresponding to albumin and the α-, β-, and γ-globulin fractions, with the γ region representing immunoglobulins. In contrast, the antibody fractions obtained after purification showed a marked enrichment of the γ-globulin region, accompanied by a substantial reduction of non-immunoglobulin proteins. The densitometry profile of the purified fraction was dominated by a single, sharp peak in the γ region, indicating efficient and specific isolation of IgG. These results confirm the successful purification and concentration of immunoglobulins, particularly in Fraction 2.

### 3.12 Evaluation of the specificity of neutralizing antibodies

**FIGURE 11:**
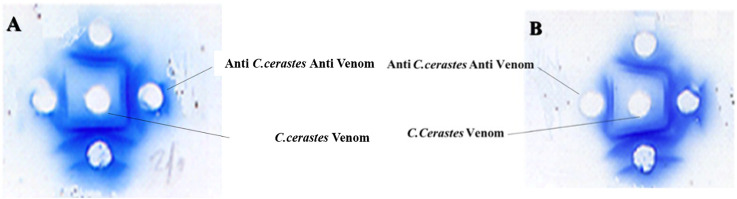
Purification and evaluation of immunoglobulin g (IgG) from plasma via densitometric analysis.

The results obtained from the Ouchterlony test, an immunodiffusion technique, demonstrate that the antivenom produced is specific to the venom of *Cerastes cerastes* (*TABL*) (Figure 11).

### 3.13 Evaluation of the efficacy of neutralizing antibodies

#### 3.13.1 Determination of the Median Effective Dose (ED50)

To assess the efficacy of the specific antivenom (*Cc*Mo_AV) against the lethal, hemorrhagic, and edematous effects of *Cerastes cerastes* venom, *in vivo* experiments were performed. Its performance was compared with Inoserp® MENA, the only commercially available antivenom in Morocco.

For the lethal activity, *Cc*MoAv exhibited strong neutralizing capacity, with an ED_50_ value of 20.11 µL (95% CI: 16.8–21.44) corresponding to a neutralization potency of 7.22 mg/mL, while Inoserp® MENA showed a higher ED_50_ of 38.13 µL (95% CI: 32.74–44.19) and a lower potency of 1.93 mg/mL, indicating greater effectiveness of *Cc*MoAv in preventing lethality (Table 4).

**TABLE 4:** Neutralization of pathological effects induced by *Cerastes cerastes* venom by the specific antivenom (*CcMo AV*).

*Cerastes cerastes venom*							
i.p LD_50_ (μg/g)	Challenge dose	Neutralization of the lethal activity					
1.815 (1.54–2.09)	5LD_50_	Antivenom	ED_50_^a^ (μL)	P^b^ (mg/mL)	Antivenom	ED_50_^a^ (μL)	P^b^ (mg/mL)
		*CcMo_AV*	20,11 (16,8–21,44)	7, 22	Inoserp-MENA	38.13 (32.74–44.19)	1.93
i.d MHD (µg)	Challenge dose	Neutralization of the hemorrhagic activity					
0,34 ± 0,11	2MHD	Antivenom	0,34 ± 0,11	2MHD	Antivenom	0,34 ± 0,11	2MHD
		*CcMo_AV*	*1.6* (1.523–1.725)	0.30 (0.266–0.385)	Inoserp-MENA	3.644 (3.193–4.179)	0.30 (0.266–0.385)
i.pl MED (µg)	Challenge dose	Neutralization of the edema-forming activity					
1,74 ± 0,82	2MED	Antivenom	1,74 ± 0,82	2MED	Antivenom	1,74 ± 0,82	2MED
		*CcMo_AV*	8.2	3.6 (2,923–4.255)	Inoserp-MENA	21.98 (18.99–25.42)	3.6 (2,923–4.255)

Regarding the hemorrhagic activity, the specific antivenom neutralized the effect with an ED_50_ of 1.6 µL (95% CI: 1.523–1.725) and a potency of 0.30 mg/mL, whereas Inoserp® MENA required a higher volume (3.644 µL, 95% CI: 3.193–4.179) to reach 50% neutralization, showing similar potency but lower efficiency.

For the edema-forming activity, CcMoAv achieved 50% inhibition at 8.2 µL (95% CI: 2.923–4.255) with a neutralization potency of 3.6 mg/mL, compared to Inoserp® MENA, which presented a higher ED_50_ of 21.98 µL (95% CI: 18.99–25.42) and an equivalent potency (3.6 mg/mL).

Overall, these findings highlight that CcMoAv displays superior neutralizing efficacy against the lethal, hemorrhagic, and edematous effects of *Cerastes cerastes* venom when compared with the polyvalent Inoserp® MENA antivenom.

*a ED50:* Median Effective Dose, defined as the dose of antivenom (µL) at which 50% of the mice survived. The 95% confidence interval is provided in parentheses. The antivenom concentration is 20 mg/mL a’ MHD-ED50: Median Effective Dose for the neutralization of hemorrhagic activity, defined as the dose of antivenom (µL) at which venom-induced hemorrhage is reduced by 50%. The concentration is 10 mg/mL a’’ MED-ED50: Median Effective Dose for the neutralization of edematous activity. The antivenom concentration is 10 mg/mL.

b P: Potency, defined as the amount of venom (mg) completely neutralized by 1 mL of antivenom.

#### 3.13.2 Neutralization of myotoxic activity

Macroscopic observations revealed a significant neutralization of myotoxic effects in the gastrocnemius muscle envenomed by *C. cerastes* venom and subsequently treated with the specific antivenom (CcMo_AV). The protective effect of the antivenom was evident in the absence of hemorrhaging and swelling compared to untreated envenomed muscles.

**FIGURE 12:**
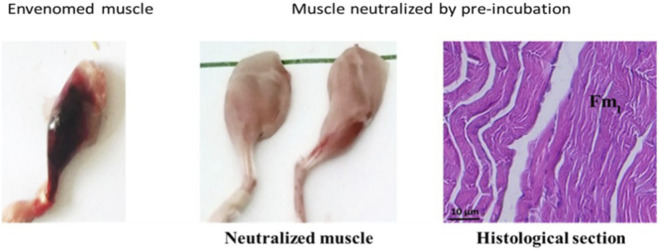
Histopathological analysis of mouse muscle following envenomation. Macroscopic and microscopic observations of mouse muscle 3 h post-envenomation. The control group exhibits extensive damage, including hemorrhage and muscle degradation. In contrast, muscle tissue treated with a specific antivenom (*Cc*Mo_Av) shows preserved structure with intact muscle fibers (Fm), indicating effective neutralization of myotoxic activity.

Histological analysis further corroborated this finding, showing no signs of myonecrosis, with complete neutralization of hemorrhage, edema, and inflammatory infiltrate. The muscle tissue appeared structurally normal, resembling the appearance of control (non-envenomed) muscles (Figure 12).

#### 3.13.3 Neutralization of tissue alterations

**FIGURE 13:**
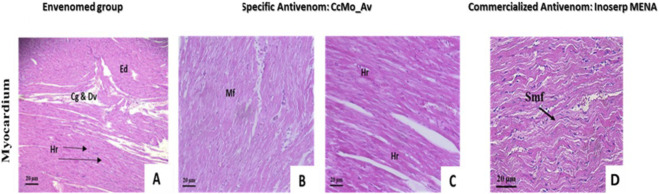
Histopathological analysis of cardiac tissue following intraperitoneal injection of Cerastes cerastes venom and treatment with specific antivenom (CcMo_AV). **(A)** Cardiac tissue from the envenomed group shows severe pathological alterations, including edema (Ed), hemorrhage (Hr), cellular degradation (Cg), and disrupted vasculature (Dv). **(B,C)** Cardiac tissue from antivenom-treated animals shows partial protection, characterized by preservation of muscle fibers (Mf) and a reduction, but not complete elimination, of hemorrhagic areas (Hr). **(D)** Cardiac tissue from the antivenomtreated group still exhibits separated and disorganized muscle fibers (Smf), indicating persistent structural alterations and incomplete neutralization of venom-induced damage.

**FIGURE 14:**
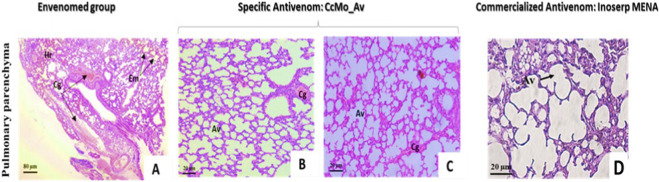
Histopathological studies of pulmonary tissues. Microscopic observations of pulmonary tissues following intraperitoneal injection of Cerastes cerastes venom and treatment with a specific antivenom (CcMo_Av) or a commercial antivenom (Inoserp MENA). **(A)** Pulmonary tissue from the envenomed group exhibits severe edema (Em), hemorrhage (Hr), and marked cellular degradation (Cg). **(B,C)** Pulmonary tissues from mice treated with the specific antivenom (CcMo_Av) show a clear restoration of alveolar architecture (Av) with minimal cellular damage, demonstrating the high efficacy of the antivenom in neutralizing venom-induced pulmonary alterations. **(D)** Pulmonary tissue treated with the commercial antivenom Inoserp MENA shows partial preservation of alveolar structures, with residual vascular alterations (V), indicating a less complete protection compared to the specific antivenom.

Tissue alterations observed in vital organs, particularly the heart (Figure 13) and lungs (Figure 14), were partially neutralized following antivenom administration, indicating a marked but incomplete resolution of severe envenomation-induced lesions. In envenomed animals, pronounced pathological changes were evident, including cardiac and pulmonary hemorrhages, bronchial vascular congestion without dilation, cardiac interstitial edema, and an emphysematous pulmonary appearance (Figures 13A, 14A).

Treatment with the specific antivenom resulted in a clear attenuation of these lesions, with reduced hemorrhagic areas and improved tissue organization in cardiac and pulmonary sections (Figures 13B, 14B,C). However, residual structural alterations persisted in some sections, reflecting incomplete neutralization of venom-induced damage (Figure 13D).

In pulmonary tissues, severe edema (Em), hemorrhage (Hr), and marked cellular degradation (Cg) were observed in the envenomed group (Figure 14A). Mice treated with the specific antivenom (*Cc*Mo_Av) showed clear restoration of alveolar architecture (Av) with minimal cellular damage (Figures 14B,C), whereas treatment with the commercial antivenom (Inoserp MENA) resulted in partial preservation of alveolar structures with residual vascular alterations (V) (Figure 14D).

These observations highlight the superior protective effect of the monovalent antivenom (*Cc*Mo_Av) compared to the commercial polyvalent formulation in mitigating the histopathological damage caused by *Cerastes cerastes* venom.

Furthermore, the antivenom exhibited substantial efficacy in preserving the normal architecture of the alveoli, with no enlargement of the alveolar spaces and intact striation of myofibers (Figures 13B,C; Figures 14B,C).

## 4 Discussion

Snakebite envenomation caused by *Cerastes cerastes* in the Maghreb region presents a serious public health challenge due to the venom’s complex biochemical composition, which includes phospholipases A2 (PLA2s) and snake venom metalloproteinases (SVMPs). These toxins contribute to severe pathological effects such as hemorrhage, edema, and myotoxicity. Despite the considerable medical burden posed by envenomation, the scarcity of specific antivenoms limits effective treatment, underscoring the urgent need for a regionally tailored antivenom (Gutiérrez and Rucavado, 2000; Serrano and Maroun, 2005). Several studies have highlighted the molecular similarities between *C. cerastes* venom from Moroccan and Tunisian populations. Previous studies demonstrated that the venom toxin profiles of these populations are closely aligned, indicating that a monovalent antivenom could be developed to neutralize venom from both regions effectively. Electrophoretic analyses confirm the conservation of key venom protein families, particularly SVMPs and PLA2s, across both populations (Calvete, 2013; Bazaa et al., 2005). The presence of dominant SVMPs in the 66–45 kDa and 21–14 kDa ranges, known for their potent hemotoxic effects, suggests that a single, well-targeted antivenom could be effective. Further toxicological assessments reveal that the venom exhibits strong hemorrhagic and edematogenic activities, with a minimal hemorrhagic dose (MHD) of 0.57 ± 0.23 μg and an edematogenic effect of 0.50 ± 0.22 μg. Additionally, histopathological examinations show that *C. cerastes* venom causes severe myonecrosis, vascular congestion, and inflammatory infiltration, emphasizing its systemic toxicity. The venom’s LD50 is approximately twice as potent via the intravenous route compared to the intraperitoneal route, highlighting the necessity of immediate medical intervention following envenomation.

To address the limitations of polyvalent antivenoms, a monovalent antivenom was developed using hyperimmune plasma from rabbits immunized with *C. cerastes* venom. The immunization process, enhanced with Freund’s adjuvants, led to the production of highly specific antibodies. Affinity chromatography purification yielded a high-concentration antibody preparation, and specificity was confirmed using immunodiffusion techniques. The developed antivenom demonstrated superior neutralization efficacy compared to commercially available polyvalent antivenom Inoserp-MENA, with a significantly lower ED50 indicating enhanced potency against *C. cerastes* venom. Although the venoms analyzed in this study were sourced exclusively from the Fask region in Morocco, which may limit geographical representativeness, and the assessment of antibody response and neutralization capacity remained qualitative without evaluation of long-term durability, the findings nonetheless provide robust evidence for the efficacy of the developed serum. Furthermore, while aspects related to industrial-scale production and the economic feasibility of a monovalent antivenom were beyond the scope of the present work, these results highlight the importance of extending future investigations to include a broader diversity of venoms in order to further optimize the neutralization spectrum of *Cc*Mo_AV.

This monovalent antivenom presents multiple advantages over polyvalent alternatives: (1) Geographical adaptation–its effectiveness across Moroccan and Tunisian *C. cerastes* populations ensures broad regional applicability; (2) Reduced dependence on imported antivenoms–local production enhances accessibility and cost-effectiveness; (3) Improved treatment outcomes–higher specificity leads to better neutralization and fewer adverse reactions compared to polyvalent antivenoms, which often contain non-specific antibodies.

Given the urgent need for effective envenomation management in North Africa, our study highlights the necessity of producing targeted antivenoms that neutralize the most clinically relevant venom components. The monovalent antivenom developed against *C. cerastes* represents a critical advancement in snakebite treatment, offering improved efficacy, reduced side effects, and enhanced accessibility for affected populations.

## Data Availability

The raw data supporting the conclusions of this article will be made available by the authors, without undue reservation.
